# Training model for the intraluminal continuous suturing technique for microvascular anastomosis

**DOI:** 10.1038/s41598-021-84619-6

**Published:** 2021-03-01

**Authors:** Zongyu Xiao, Madjid Samii, Ji Wang, Qi Pan, Zhimin Xu, Hu Ju

**Affiliations:** 1 Department of Neurosurgery, International Neuroscience Institute, 30625 Hannover, Germany; 2 Department of Neurosurgery, Affiliated Hospital of Qinghai University, Xining, 810000 China; 3 Department of Neurosurgery, The Second Affiliated Hospital of Soochow University, Suzhou, 215004 China; 4 Department of Neurosurgery, Affiliated Hospital of Hainan Medical College, Haikou, 570100 China; 5 Department of Neurosurgery, The Seventh Medical Center of PLA General Hospital, Beijing, 100000 China

**Keywords:** Experimental models of disease, Translational research, Cerebrovascular disorders, Stroke

## Abstract

Microvascular anastomosis is a critical procedure in cerebral bypass surgeries. In some rare cases, the extraluminal interrupted technique is not optimal because the vessels are immobile and cannot be rotated, and anastomosis can be performed effectively through the intraluminal continuous suturing technique. The authors reported the application of the intraluminal continuous suturing technique in microanastomosis training with silicone tube, rat’s common iliac arteries and abdominal aorta. A silicone tube with a diameter of 1.5 mm was used to practice microanastomosis in intraluminal continuous suturing technique. Then the technique was applied in side-to-side, end-to-side anastomoses of common iliac arteries and the end-to-end abdominal aorta anastomoses of rat. The suturing time and patency rates were compared with an alternative intraluminal continuous suturing technique and one-way-up interrupted suturing technique in silicone tube and rat vessel anastomoses. The intraluminal continuous suturing technique could be gained through practicing with silicone tube, and the technique has also been demonstrated effective in side-to-side, end-to-side anastomoses of common iliac arteries of rat and the abdominal aorta end-to-end anastomoses. In all the animal experimental groups with different suturing techniques, there was no difference between the patency rates, all the immediate patency rate was 100%. There was no significant suturing time difference between the two intraluminal continuous suturing techniques, but the two intraluminal continuous suturing techniques were faster than the interrupted technique. The intraluminal continuous suturing technique described in the study could be used as an efficient method for side-to-side, end-to-side and end-to-end anastomosis, especially under the situation the posterior wall of the anastomosis could not be rotated. Proficiency of the technique could be achieved through practicing in laboratory with silicone tube and live animals.

## Introduction

Microvascular anastomosis is a critical procedure in cerebral bypass surgeries^1–4^. Regardless of how complex the bypasses are, they are all built with three simple anastomoses: end-to-side, end-to-end, and side-to-side^5^. Traditionally, anastomoses should be performed with the extraluminal interrupted technique, and the surgery must be performed under little or no tension to connect the vessels; however, in some rare situations, the extraluminal interrupted technique is not optimal because the vessels are immobile and cannot be rotated^6–9^, and anastomosis can be performed effectively through the intraluminal continuous suturing technique, which is also called the “in situ suturing technique”. The intraluminal continuous suturing technique does not require the vessel to be rotated, the posterior wall of the anastomoses should be sutured in an intraluminal fashion after placing the first two anchoring stitches, and the anterior wall can be easily closed in the traditional way; this technique has been effectively used as a standard technique for side-to-side anastomoses^2,8,10,11^, but it can also be applied in end-to-end and end-to-side anastomoses. In this article, we described a training model for the intraluminal continuous suturing technique with silicone tubes and showed the technique in side-to-side, end-to-side and end-to-end anastomoses in rats. The suturing time and patency rates were compared with an alternative intraluminal continuous suturing technique and one-way-up interrupted suturing technique in silicone tube and rat vessel anastomoses.

## Results

In microsurgical suturing training with silicone tubes, an approximately 4.5 mm linear incision was made on both the donor and recipient silicone tubes for side-to-side anastomosis. In addition to two anchoring sutures, approximately 12–15 sutures were needed to close the anterior or posterior wall. For end-to-side anastomosis, a 1.5 mm arteriotomy was performed, and an average of 8–10 sutures were placed intraluminally for the posterior wall and extraluminally for the anterior wall of the anastomoses. For end-to-end anastomosis, the diameter of the silicone tube was approximately 1.5 mm, and approximately 8–10 intraluminal sutures and 8–10 extraluminal sutures were placed to complete the anastomoses.

In the animal experiments, the diameters of the common iliac arteries (CIAs) and abdominal aorta were approximately 1 mm and 2 mm, respectively. For side-to-side anastomoses between CIAs, the arteriotomy was approximately 3 mm, and 12–15 sutures were needed to close the posterior wall or the anterior wall. For end-to-side anastomoses using the CIAs, the arteriotomy was approximately 3 mm, and 12–15 sutures were needed to close each side of the wall. For end-to-end anastomoses using the abdominal aorta, approximately 6–8 sutures were needed for each side of the wall. No animal deaths were recorded during the procedure in this study.

In all the animal experimental groups with different suturing techniques, no thrombus was observed at the anastomosis site, pulsation of the recipient artery distal to the anastomosis was observed after releasing the clips in all anastomoses under a microscope (Video 1), and Acland’s test further confirmed the patency. In the study, there was no difference between the patency rates in all different groups; all the immediate patency rates were 100% for side-to-side and end-to-side anastomosis of the CIAs and end-to-end anastomosis of the abdominal aorta, and all the overall patency rates were 100% thirty minutes after the restoration of blood flow.

**Figure 1:**
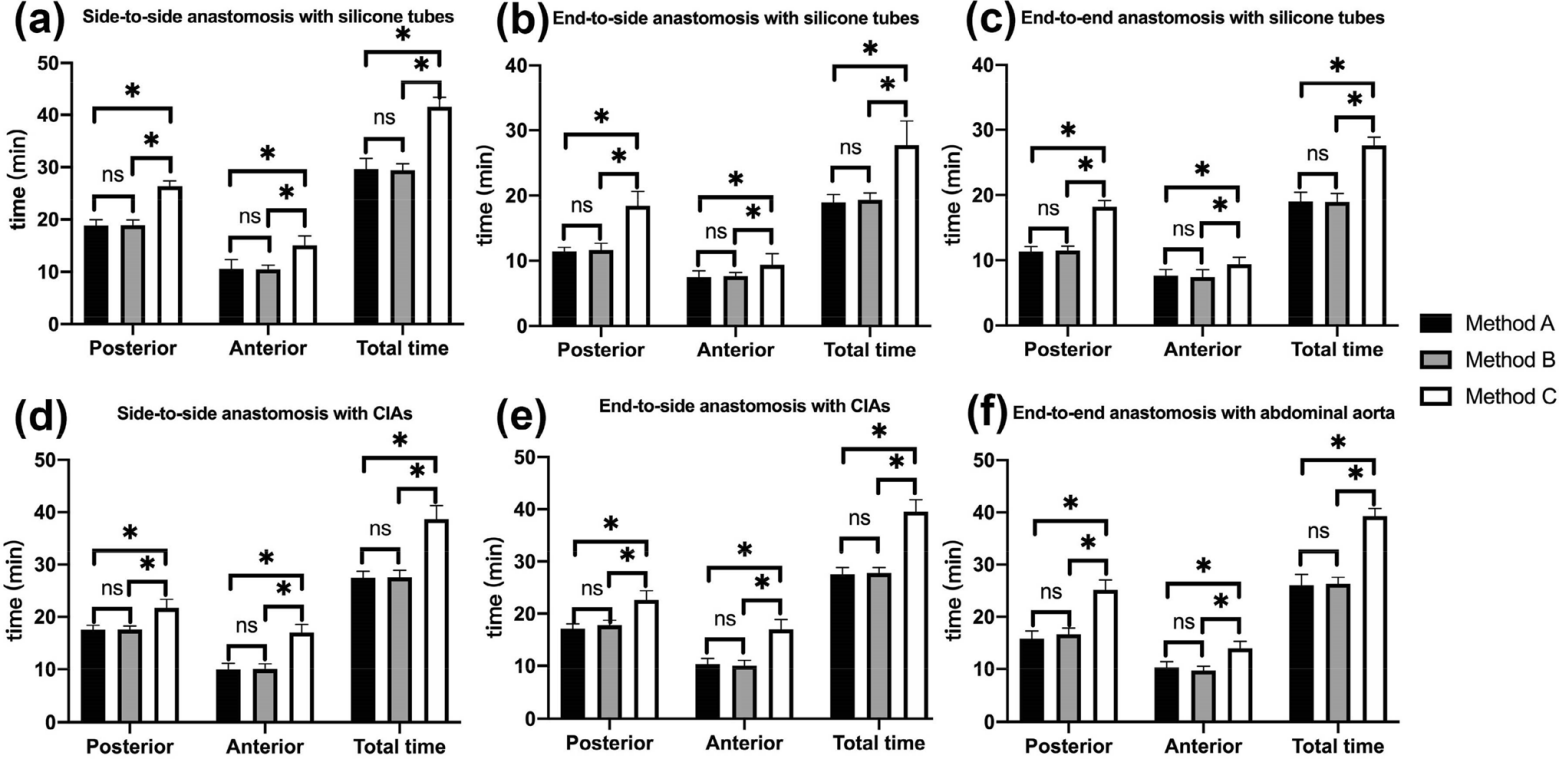
Comparison of the suturing time with silicone tubes (**a**–**c**) and rat vessels (**d**–**f**) with three methods in different anastomoses. The average total suturing time (Total), respective suturing time for posterior wall (Posterior) and anterior wall (Anterior) in different anastomoses with silicone tubes and rat vessels were analyzed by one-way ANOVA. In all the groups, Method A and Method B were faster than Method C in total suturing time, respective suturing time for posterior and anterior wall. There was no significant difference between Method A and Method B in suturing times. Method A: the intraluminal continuous suturing technique, Method B: the alternative intraluminal continuous suturing technique, Method C: one-way-up interrupted suturing technique.

In all the groups with three different suturing methods, regardless of suturing with silicone tubes or rat vessels, the suturing time for posterior walls was significantly longer than that of anterior wall in each side-to-side, end-to-side and end-to-end anastomosis. There was no significant difference in the total suturing time or the respective suturing time for the posterior and anterior walls between the intraluminal continuous suturing technique Method A and the alternative intraluminal continuous suturing technique Method B in all different anastomoses. Compared with the one-way-up interrupted suturing technique Method C, Method A and Method B were faster than Method C in total suturing time, respective suturing time for posterior and anterior wall in silicone tubes and rat vessels anastomoses (Fig. 1, the details of the average total suturing time, and respective suturing time for posterior and anterior walls with silicone tubes and rat vessels were seen in supplementary Table S1, and supplementary Table S2).

## Discussion

The ability to create patent microvascular anastomoses is a critical procedure in cerebral bypass surgeries^1–4^. Yasargil often emphasized the absolute necessity of laboratory training in microtechniques before these methods are applied to patients in the operating room^9^. Therefore, before the clinical application of cerebral revascularization, undergoing plenty of microsurgical training in the laboratory to become familiar with anastomosis procedures is fundamental, and the technique can be learned through deliberate practice in microsurgical laboratories.

Several training methods for microvascular anastomosis with gauze, silicone tubes, chicken wings, human placenta, cadaveric brains and some animal models (such as rats and mice) have been described previously^12–15^. Even though silicone tubes do not have dynamic circulation and are different from human vessels, these tubes also have several advantages: they are convenient and cheap, they can be easily used without any special technique for maintenance or conservation, and there are no ethical concerns. Therefore, silicone tubes are more suitable for beginners to learn the basic microsurgical technique and allow the trainee to periodically practice, even at the office or at home. However, anastomotic patency cannot be evaluated during microsurgical training with silicone tubes^13^. Therefore, it is suggested that further microanastomosis training should be conducted in living animals after the basic microvascular anastomosis techniques are mastered with nonliving models. Rats are the most commonly used animal for microsurgical training, and the vessels of rats can mimic real anastomotic surgery in terms of texture, haptic qualities, pulsation and coagulation physiology. When practicing with rat vessels, all manipulations must be performed as precisely and gently as possible, and these vessels are very useful for trainees to learn how to remove the adventitia. The patency of the anastomoses can be evaluated immediately after the anastomoses are complete. All of these advantages cannot be reproduced in nonliving models, which makes the living animal model very important before applying the technique in a real human body^9,16^.

Regardless of how complex bypass surgery is, there are only three types of anastomoses: end-to-side, end-to-end, and side-to-side^5^. Traditionally, the majority of anastomoses are performed extraluminally in an interrupted fashion, and the vessels should be placed with little or no tension. However, in some rare cases, the artery cannot be rotated to fully expose the posterior wall for the anastomoses due to the short stump, important perforators, or deep and narrow surgical corridor^6–9^. In this situation, the traditional extraluminal interrupted suturing technique could be very difficult to perform on the posterior wall. The intraluminal continuous suturing technique is an effective method to solve this problem and can close the posterior wall without rotating the vessel.

In this study, we used a silicone tube to start the training for the intraluminal continuous suturing technique. Regardless of the type of anastomosis, the suturing technique is almost always the same, and only the size of the anastomoses is different. The appropriate length of the sutures should be chosen according to the size of the arteriotomy before the procedure. The first anchoring suture was placed traditionally, but the tail of the suture was left in place to the knot later. After the second anchoring suture was placed, the needle and the tail of the suture were kept in place; the suture was inserted into the vessel from the outside to the inside, and the posterior wall was sutured between the anterior wall of the arteries. Then, the needle exited the vessel, the suture was tied with the tail of the first anchoring suture, and the anterior wall was closed in an extraluminal continuous suturing fashion with the same needle. When the trainee became familiar with the intraluminal continuous suturing technique with a silicone tube, it is suggested that the trainee should begin to practice the technique on living animals. In this study, the intraluminal continuous suturing technique was also successfully applied in side-to-side and end-to-side anastomoses between CIAs and end-to-end anastomoses with the abdominal aorta. Mastering the technique in the laboratory is the key prerequisite before applying it in patients. Currently, the intraluminal continuous suturing technique has been successfully used in anterior cerebral artery (ACA)-ACA, posterior inferior cerebellar artery (PICA)-PICA, PICA-anterior inferior cerebellar artery (AICA), posterior cerebral artery (PCA)-superior cerebellar artery (SCA), middle cerebral artery (MCA)-MCA, and superficial temporal artery (STA)-MCA side-to-side anastomoses^2,8^.

In addition to the intraluminal continuous suturing technique, there are still some other techniques for suturing the posterior wall when the vessel cannot be rotated. A segment of an interposition vessel graft could be used to increase the length of the vessel, which could make the vessel much easier to rotate and enable suturing under a traditional extraluminal suturing fashion, but this method needs to harvest a segment of matched artery or vein graft, and the procedure composed of at least two anastomoses, so it is time-consuming and could significantly increase the ischemic risk^17^. Cigna^18^ used the posterior-wall-first interrupted suturing technique to close the posterior wall of a small vessel with 3 sutures as follows: the first suture was placed in the middle of the posterior wall, and then the second and third sutures were placed around the first stitch, one above and one below, but this method with 3 sutures was only applicable in small vessels. In this study, we compared our method with two other types of suturing techniques, the alternative intraluminal continuous suturing technique Method B reported by Ramanathan^10^ and the one-way-up interrupted suturing technique Method C as described by Pruthi^19^ and Bas^20^. Method C is actually another practical way to suture the posterior wall when the vessel cannot be rotated. Our results showed that there was no difference between the anastomosis patency rates of Method C compared to the intraluminal continuous suturing technique Method A and Method B, but Method C requires a knot after every stitch, which was obviously time-consuming compared to Method A and Method B in total suturing time and the respective suturing time for posterior and anterior walls. In the groups with Method B, there was no significant difference between the suturing time and anastomosis patency rates compared with Method A. Only a staying suture was needed in the first step of Method B, which may expand the working space between the two vessels during closure of the posterior wall and help identify the posterior wall, but this method may also increase the risk of vessel tearing due to the tension between the vessels. In Method A, we sutured the posterior wall with an intraluminal continuous suturing technique after the first two anchoring sutures were placed, which could help hold the posterior wall in place and reduce the tension between the arteries. Harashina^21^ sutured the posterior wall of a rat vessel using a continuous suture without knotting. Then, two interrupted sutures followed on both sides of the arteriotomy, and the ends of the continuous suture were tied with the interrupted suture, but they closed the anterior wall in a standard interrupted suturing technique to complete the anastomoses. Comparing the suturing time in the study, whether suturing with the intraluminal continuous suturing technique or the interrupted suturing technique, our results demonstrated that the posterior wall needs more time to be closed with the same suturing technique due to the narrow working space between the anterior walls. Furthermore, our results showed that the intraluminal continuous suturing techniques Method A and Method B were faster than the interrupted suturing technique Method C in the total suturing time and respective suturing time for anterior or posterior walls, so it is suggested that Method A and Method B would be faster than Harashina’s technique. All of these techniques described above were very useful to close the posterior wall, but the surgeon should apply these methods based on the real surgical situation and their dexterity and experience.

However, there were still some drawbacks in our study. We only demonstrated the application of the intraluminal continuous suturing technique with a 1.5 mm diameter silicone tube and rat CIA and abdominal aorta, but we believe that trainees could use this technique on different sizes of silicone tubes and real vessels. We recommended that trainees also undergo in-depth training with this technique to mimic the procedure in a real deep, narrow surgical corridor. Although the intraluminal continuous suturing technique is more challenging than some other methods, this technique would be a valuable tool for revascularization in cerebrovascular surgery.

## Conclusions

The intraluminal continuous suturing technique described in the study could be used as an efficient method for side-to-side, end-to-side and end-to-end anastomoses, especially when the posterior wall of the target vessel cannot be rotated. Proficiency of the technique can be achieved through deliberate practice in the laboratory with silicone tubes and live animals.

## Methods and materials

A silicone tube with a diameter of 1.5 mm was supplied by Professor Yi Wang of the Affiliated Drum Tower Hospital, Nanjing University. 180 male Sprague–Dawley (SD) rats weighing 200–250 g were used in the study. A training microsurgical microscope (Zeiss, OPMI Pico), 10–0 monofilament nylon sutures, microforceps with tips of 0.15 mm and 0.3 mm, microscissors and microneedle holders were used in the study. All procedures were performed by the first author under 10 × or 16 × magnification under a microscope. In animal experiments, patency was observed by direct observation under the microscope after the blood flow was restored immediately and 30 min later.

### Ethical statement

The study was carried out in compliance with the ARRIVE guidelines, and all animal experiments were performed according to national and international guidelines and were approved by the Research Ethics Committee of the Affiliated Hospital of Qinghai University. This article does not contain any studies with human participants performed by any of the authors.

### Microanastomosis training with silicone tubes

**Figure 2:**
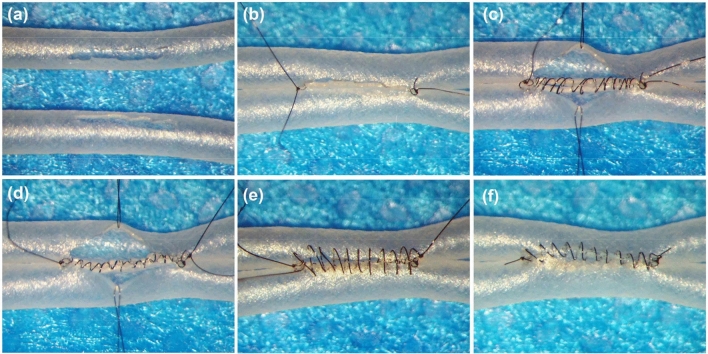
Side-to-side anastomosis by using silicone tube. Longitudinal incisions of approximately three times the diameter of the silicone tube were made on both silicone tubes (**a**). Two 10–0 anchoring sutures were placed at opposite ends of the anastomoses (**b**). The posterior wall was closed intraluminally in a loose continuous suturing fashion (**c**) and these sutures were tightened (**d**). The anterior wall was closed extraluminally in a loose continuous suturing technique (**e**) and these sutures were tightened (**f**).

**Figure 3:**
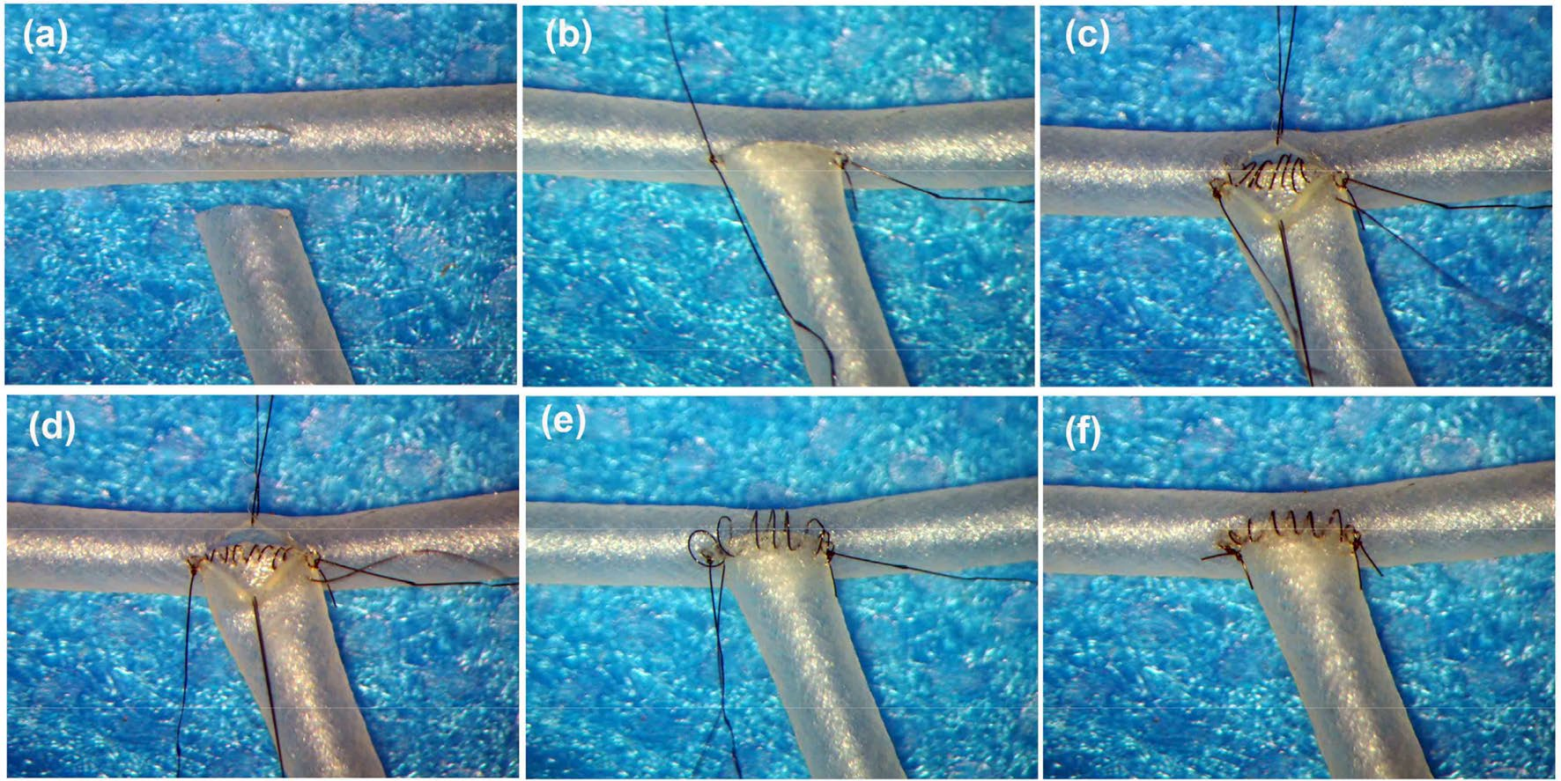
End-to-side anastomosis by using silicone tube. The end of the donor silicone tube was trimmed, and a same size of longitudinal incision was performed on the recipient silicone tube (**a**). The heel and toe stitches were placed (**b**). The posterior wall was closed intraluminally in a loose continuous suturing technique (**c**) and these sutures were tightened (**d**). The anterior wall was closed extraluminally in a loose continuous suturing fashion (**e**) and these sutures were tightened (**f**).

**Figure 4:**
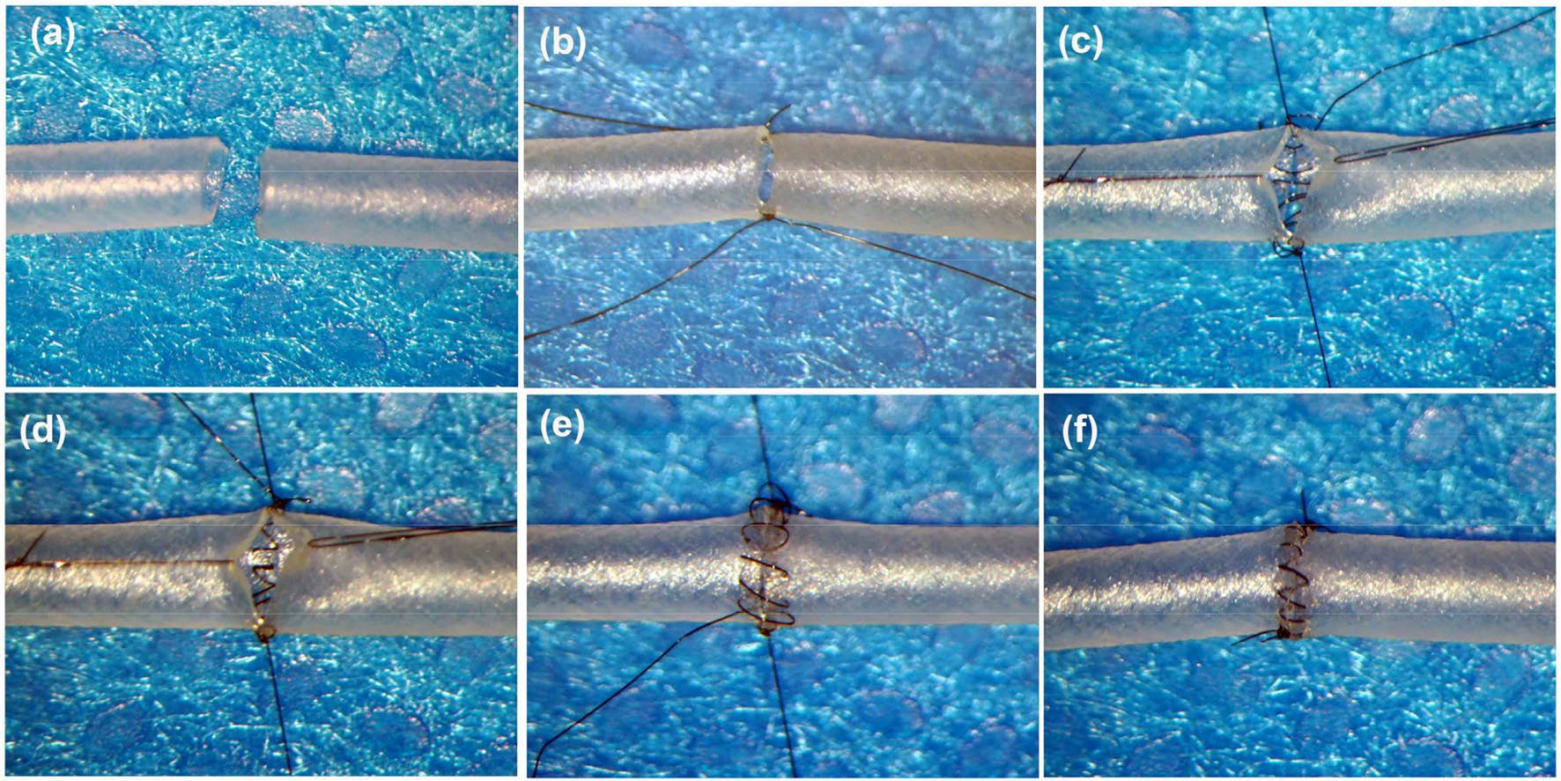
End-to-end anastomosis by using silicone tube. Both ends of the silicone tube was trimmed (**a**). Two anchoring sutures were placed (**b**). The posterior wall was closed intraluminally in a loose continuous suturing technique loosely (**c**) and tightened (**d**). The anterior wall was closed extraluminally in a loose continuous suturing fashion (**e**) and these sutures were tightened (**f**).

For side-to-side anastomosis, two silicone tubes were placed parallel next to each other, and longitudinal incisions of approximately three times the diameter of the silicone tube were made on both silicone tubes (Fig. 2a). Two 10–0 anchoring sutures were placed at the opposite ends of the anastomoses to join the silicone tubes together, and the tail of the sutures and one needle with an appropriate length of suture were kept in place (Fig. 2b). Then, the needle was inserted into the first silicone tube from outside to inside just under the first anchoring knot, and the posterior wall was closed intraluminally in a loose continuous suturing fashion. The depth of the sutures was one to two times the wall thickness, and the spacing was approximately three to five sutures per millimeter. After all the sutures were placed intraluminally, the needle was passed through the posterior wall of the silicone tube from inside to outside just under the second anchoring knot. Then, the loose spiral of the intraluminal sutures was tightened one by one from beginning to the end, and the suture was tied to the tail of the other anchoring knot. Finally, the anterior wall of the silicone tube was closed in the same continuous fashion (Fig. 2c–f, Video 2). The same technique could also be used for end-to-side and end-to-end anastomoses (Figs. 3 and 4, Videos 3–4).

### Side-to-side anastomosis of rat common iliac arteries

**Figure 5:**
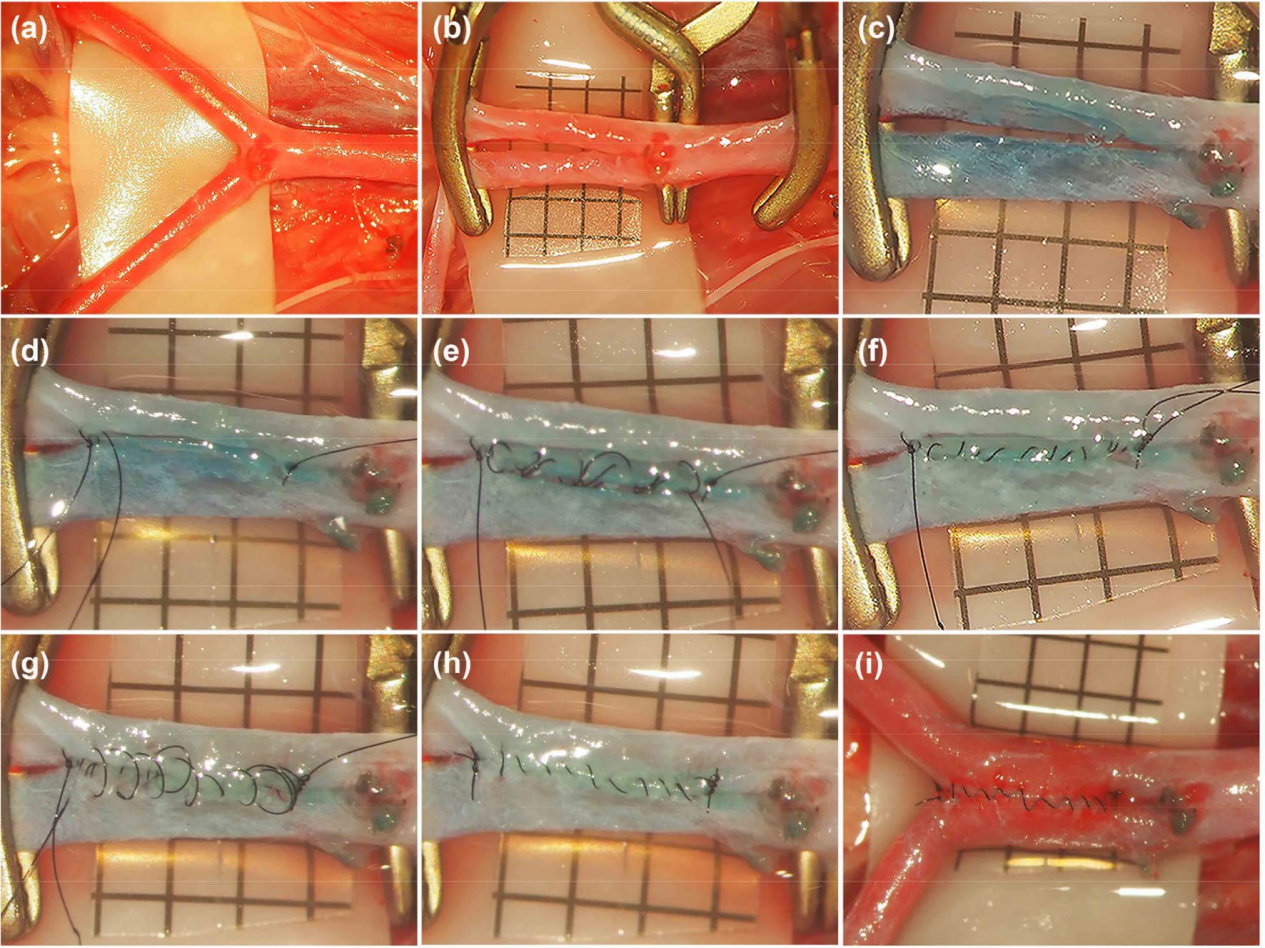
Side-to-side anastomosis of bilateral common iliac arteries. After both common iliac arteries were dissected (**a**), the caudal artery was temporarily clipped, the bilateral common iliac arteries were drawn close to each other and temporarily clipped (**b**). Arteriotomies of approximately 3 times the diameter of the common iliac artery were performed on both arteries (**c**), two anchoring stitches were placed on both opposite side of the anastomoses (**d**), the posterior wall was closed intraluminally in a loose continuous suturing technique (**e**) and these sutures were tightened (**f**), and the anterior wall was closed extraluminally in a loose continuous suturing technique (**g**) and these sutures were tightened (**h**), and the blood flow was restored (**i**) after the temporary clips were removed. (Scale bar = 1 mm).

Twenty male SD rats were used in the study. After the rats were anaesthetized intraperitoneally with pentobarbital (50 mg/kg), they were placed in the supine position, and a midline abdominal skin incision was made from the xiphoid process to the pubic symphysis. The distal portion of the abdominal aorta and both sides of the common iliac arteries (CIAs) were carefully dissected (Fig. 5a). The caudal artery arising from the posterior aspect of the abdominal aortic bifurcation was dissected and temporarily clipped, and other branches were coagulated and cut. Then, two temporary clips were applied: one was placed at the distal segment of the abdominal aorta, and both iliac common arteries were drawn close to each other and temporally clipped by the other temporary clip (Fig. 5b). A beveled 25-gauge needle was used to initiate perforation of the iliac arteries. Then, microscissors were used to extend the arteriotomy; arteriotomies of approximately 3 times the diameter of the iliac artery were made on both iliac common arteries, the lumens of the vessels were irrigated with heparinized saline (100 units/mL), and blue dye was used to help visualize the edges of the arteriotomies (Fig. 5c). Next, 10–0 nylon was used for suturing. The same steps from the training protocol with silicone tubes were then performed. The adventitia around the arteriotomies was carefully removed. Two interrupted anchoring sutures were placed opposite to the anastomosis sites first. Then, a suture was inserted into the lumen of the artery from the outside, and the posterior wall was closed intraluminally in a continuous fashion; thus, the suture existed under the other anchoring knot and was tied with the tail of said anchoring knot. The anterior wall was also sutured extraluminally in a continuous fashion. After the anastomoses were finished, the distal clip was removed to allow blood to flow back to the anastomoses; bleeding points were gently covered with small pieces of gel foam, and then the clip on the abdominal aorta was removed to restore blood flow (Fig. 5d–i). If there was still some bleeding, several interrupted sutures were added to stop the bleeding with or without temporary clips.

### End-to-side anastomosis between the bilateral common iliac arteries

**Figure 6:**
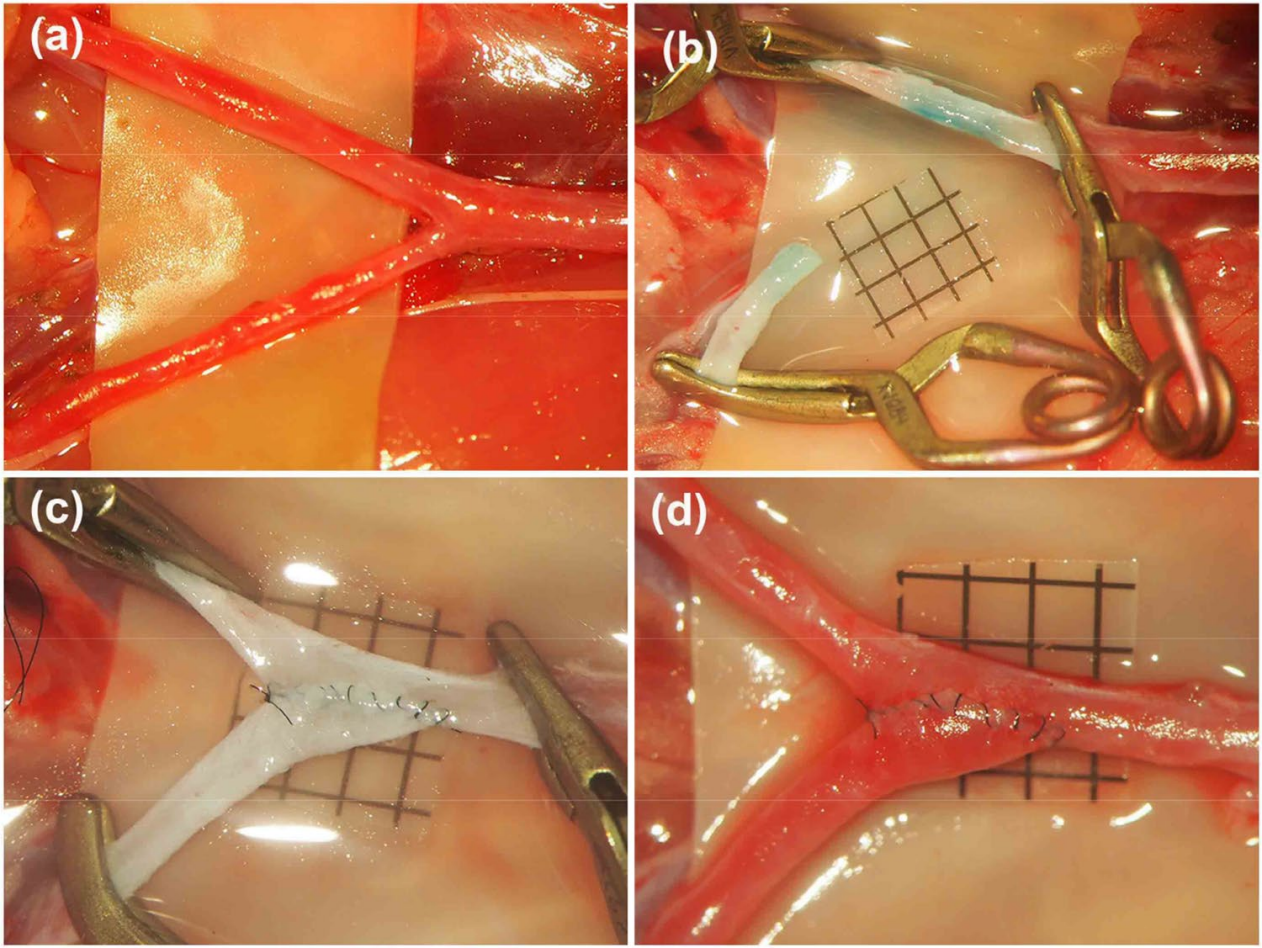
End-to-side anastomosis between bilateral common iliac arteries. After both common iliac arteries were carefully dissected (**a**). The left common iliac artery was temporarily clipped and cut at its origin from the abdominal aorta, the right common iliac artery was also temporarily clipped (**b**). The end-to-side anastomosis was completed with the intraluminal continuous suturing technique (**c**) and the blood flow was restored (**d**). (Scale bar = 1 mm).

Twenty SD rats were used in this group. After the rats were anesthetized, a midline abdominal incision was performed, and the bilateral iliac arteries were carefully dissected (Fig. 6a). A temporary clip was placed at the distal part of the left CIA, and the artery was ligated and cut at its origin from the abdominal aorta. The end of the left CIA was cut in a fish-mouth fashion, and a linear arteriotomy of the same length was made on the right CIA after it was temporarily clipped (Fig. 6b). Two anchoring sutures were placed, the posterior wall was closed in an intraluminal continuous suturing fashion, and the anterior wall was closed in an extraluminal continuous fashion. Then, blood flow was restored after the temporary clips were removed (Fig. 6c,d), and patency was evaluated.

### End-to-end anastomosis using the abdominal aorta

**Figure 7:**
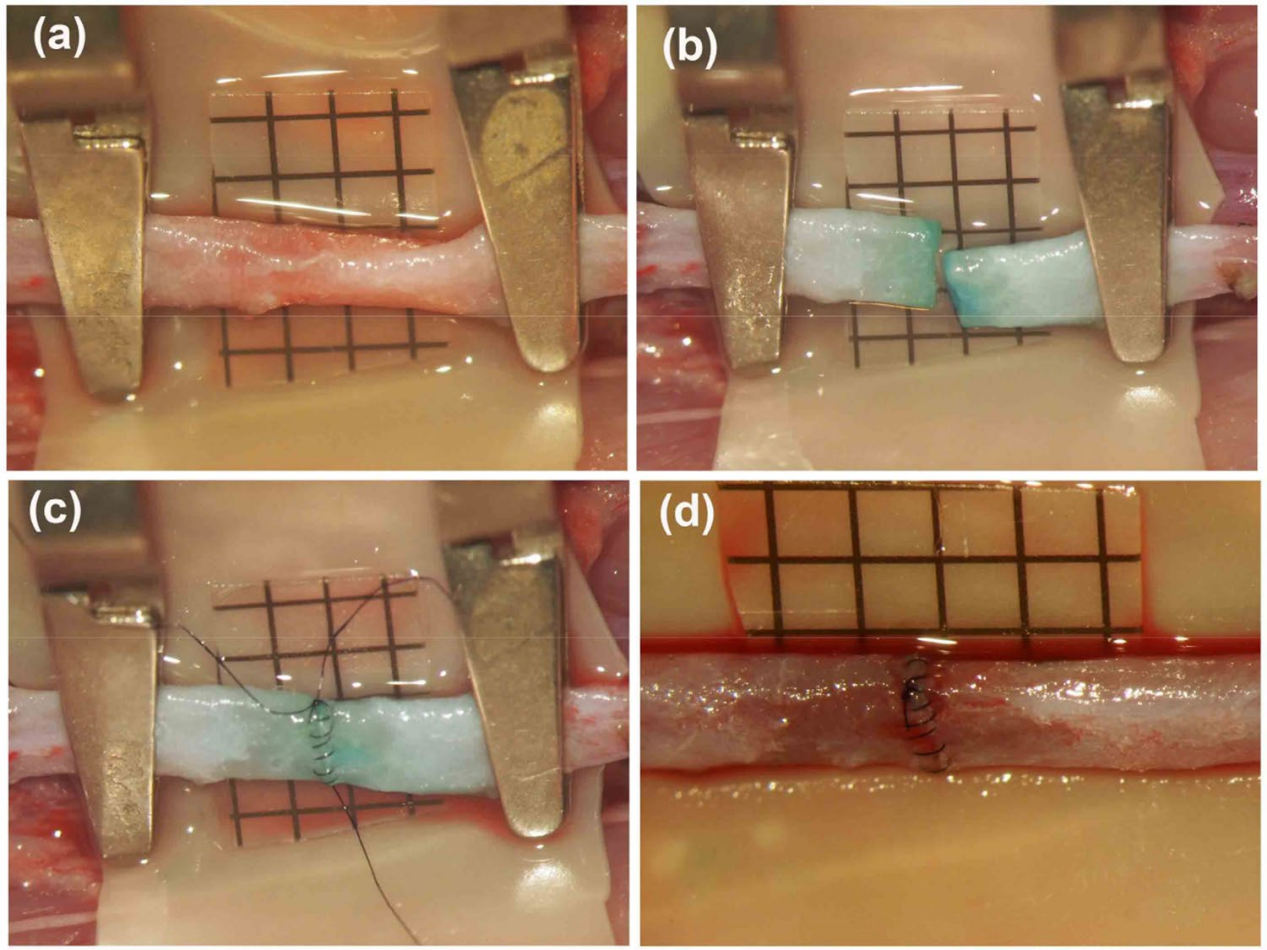
End-to-end anastomosis using abdominal aorta. After the abdominal aorta has been dissected (**a**), it was temporarily clipped and transected (**b**). The end-to-end anastomosis was completed with the intraluminal continuous suturing technique (**c**) and the blood flow was restored (**d**). (Scale bar = 1 mm).

Twenty SD rats were used in this group. The abdominal aorta was carefully dissected after a midline abdominal skin incision was performed, the abdominal aorta was temporarily clipped and transected, and end-to-end anastomosis was performed in the same intraluminal continuous fashion as mentioned above (Fig. 7).

### Comparing the suturing time and patency rates among three types of anastomoses

Three types of anastomoses were tested in the study: (1) Method A: the intraluminal continuous suturing technique as described in the study, (2) Method B: an alternative intraluminal continuous suturing technique reported by Ramanathan^10^, one anchoring suture was made in the inferior corner of the arteriotomy, the posterior wall was closed in a continuous intraluminal suturing fashion to the upper corner of the arteriotomy, where a new suture was placed in the upper corner after the complete closure of the posterior wall; the tail of the knot was then tied with the first anchoring suture. Next, another new suture may be started from the inferior corner, and both of the new sutures closed the anterior wall towards each other in an extraluminal continuous fashion and were tied together in the middle of the anterior wall. (3) Method C: One-way-up interrupted suturing technique as described by Pruthi^19^ and Bas^20^. The first staying suture was placed at the distal end of the arteriotomy, and the back wall was sutured without rotating the vessel. First, the microneedle was inserted into the first vessel from the outside and then continued to go inside to outside of the second vessel, and the knot was tied outside of the vessel. After the posterior wall was sutured, the second staying suture on the proximal end of the artery was placed, and the anterior wall was sutured with the traditional extraluminal suturing technique.

Twenty procedures of each side-to-side, end-to-side and end-to-end anastomosis with silicone tubes and corresponding rat vessels as described above were performed with the three methods. The total suturing time and the respective suturing time for the posterior and anterior walls were recorded. The patency of these anastomoses with rat vessels was accessed immediately and 30 min after releasing the clips through direct observation under a microscope and Acland’s test, which was carried out by milking the recipient artery with microforceps to empty and refill it with bypass flow. These rats were then euthanized by intraperitoneal injection of pentobarbital (200 mg/kg).

### Statistical analysis

The data are presented as the means ± standard deviation (SD). The analysis was carried out using SPSS 19.0 (SPSS, Inc., Chicago, IL, USA). Differences between groups were analyzed by one-way analysis of variance (ANOVA). A value of P less than 0.05 was considered statistically significant.

## Supplementary Information

Supplementary Tables.

Supplementary Video 1.

Supplementary Video 2.

Supplementary Video 3.

Supplementary Video 4.

## Abbreviations

SD: Sprague–Dawley
CIAs: Common iliac arteries
ACA: Anterior cerebral artery
PICA: Posterior inferior cerebellar artery
AICA: Anterior inferior cerebellar artery
PCA: Posterior cerebral artery
SCA: Superior cerebellar artery
MCA: Middle cerebral artery
STA: Superficial temporal artery
