# Supplementary material for: Training model for the intraluminal continuous suturing technique for microvascular anastomosis
Source: Sci Rep. 2021 Mar 1;11:4862. doi: 10.1038/s41598-021-84619-6 (PMC7921418; doi:10.1038/s41598-021-84619-6)
Supplement: Supplementary file 1 — Supplementary Tables. [file 41598_2021_84619_MOESM1_ESM.docx]

**Training model for the intraluminal continuous suturing technique for microvascular anastomosis**

Zongyu Xiao, Madjid Samii, Ji Wang, Qi Pan, Zhimin Xu, Hu Ju

**Zongyu Xiao**: xiaozongyu@hotmail.com, Department of Neurosurgery, International Neuroscience Institute, Hannover, 30625, Germany. Department of Neurosurgery, Affiliated Hospital of Qinghai University, Xining, 810000, China.

**Madjid Samii**: [samii@ini-hannover.de](mailto:samii@ini-hannover.de), Department of Neurosurgery, International Neuroscience Institute, Hannover, 30625, Germany.

**Ji Wang**: [MD_WangJ@163.com](mailto:MD_WangJ@163.com), Department of Neurosurgery, The Second Affiliated Hospital of Soochow University, Suzhou, 215004, China.

**Qi Pan**: [doctorpanqi@aliyun.com](mailto:doctorpanqi@aliyun.com), Department of Neurosurgery, Affiliated Hospital of Hainan Medical College, Haikou, 570100, China.

**Zhimin Xu:** [xuzhiminsjwk@163.com](mailto:xuzhiminsjwk@163.com), Department of Neurosurgery, The Seventh Medical Center of PLA General Hospital, Beijing, 100000, China.

**Hu Ju**: [geassfancy@163.com](mailto:geassfancy@163.com), Department of Neurosurgery, Affiliated Hospital of Qinghai University, Xining, 810000, China.

**Table 1 Suturing time with silicon tubes in three suturing methods (Mean ± Standard deviation)**

| Method | Side-to-side anastomoses (n=20) | | |  | End-to-side anastomoses (n=20) | | |  | End-to-end anastomoses (n=20) | | |
| --- | --- | --- | --- | --- | --- | --- | --- | --- | --- | --- | --- |
|  | Posterior | Anterior | Total |  | Posterior | Anterior | Total |  | Posterior | Anterior | Total |
| Method A | 18.85±1.13min | 10.60±1.75min | 29.65±2.03min |  | 11.45±0.60min | 7.50±0.95min | 18.95±1.19min |  | 11.35±0.75min | 7.65±0.93min | 19.00±1.41min |
| Method B | 18.95±1.00min | 10.45±0.82min | 29.40±1.27min |  | 11.65±1.04min | 7.65±0.58min | 19.30±1.08min |  | 11.50±0.69min | 7.45±1.10min | 18.95±1.31min |
| Method C | 26.40±0.99min | 15.05±1.79min | 41.55±1.82min |  | 18.40±2.23min | 9.35±1.72min | 27.70±3.70min |  | 18.20±0.95min | 9.40±1.04min | 27.60±1.27min |
| F | 343.144 | 58.68 | 319.014 |  | 145.887 | 15.03 | 90.437 |  | 474.793 | 21.757 | 277.829 |
| P-Value | 0.000 | 0.000 | 0.000 |  | 0.000 | 0.000 | 0.000 |  | 0.000 | 0.000 | 0.000 |

The average total suturing time (Total), respective suturing time for posterior wall (Posterior) and anterior wall (Anterior) in different anastomoses with silicone tubes were analyzed by one-way ANOVA. In all the groups, Method A and Method B were faster than Method C in total suturing time, respective suturing time for posterior and anterior wall. There was no significant difference between Method A and Method B in suturing times. Method A: the intraluminal continuous suturing technique, Method B: the alternative intraluminal suturing technique, Method C: one-way-up interrupted suturing technique.

**Table 2 Suturing time with rat vessels in three suturing methods (Mean ± Standard deviation)**

| Method | Side-to-side anastomoses  with bilateral common iliac arteries (n=20) | | |  | End-to-side anastomoses  with bilateral common iliac arteries (n=20) | | |  | End-to-end anastomoses  with abdominal aorta (n=20) | | |
| --- | --- | --- | --- | --- | --- | --- | --- | --- | --- | --- | --- |
|  | Posterior | Anterior | Total |  | Posterior | Anterior | Total |  | Posterior | Anterior | Total |
| Method A | 17.50±0.89min | 9.95±1.19min | 27.45±1.28min |  | 17.15±0.93min | 10.35±1.09min | 27.50±1.36min |  | 15.75±1.52min | 10.30±1.08min | 26.05±2.06min |
| Method B | 17.55±0.69min | 10.00±1.02min | 27.55±1.36min |  | 17.75±0.97min | 10.00±1.07min | 27.75±1.12min |  | 16.60±1.27min | 9.70±0.80min | 26.30±1.26min |
| Method C | 21.70±1.66min | 17.00±1.55min | 38.70±2.58min |  | 22.60±1.79min | 17.01±1.88min | 39.55±2.26min |  | 25.20±1.85min | 13.95±1.36min | 39.30±1.42min |
| F | 86.991 | 201.764 | 248.152 |  | 107.057 | 162.510 | 347.180 |  | 222.949 | 86.973 | 438.425 |
| P-Value | 0.000 | 0.000 | 0.000 |  | 0.000 | 0.000 | 0.000 |  | 0.000 | 0.000 | 0.000 |

The average total suturing time (Total), respective suturing time for posterior wall (Posterior) and anterior wall (Anterior) in different anastomoses with rat vessels were analyzed by one-way ANOVA. In all the groups, Method A and Method B were faster than Method C in total suturing time, respective suturing time for posterior and anterior wall. There was no significant difference between Method A and Method B in suturing times. Method A: the intraluminal continuous suturing technique, Method B: the alternative intraluminal suturing technique, Method C: one-way-up interrupted suturing technique.

**Video 1** Pulsation of the recipient artery distal to the anastomosis was observed under a microscope after releasing the clips in all anastomoses in intraluminal continuous suturing technique. Note that the end-to-end anastomosis was performed with double fish-mouthed arteriotomies to create a wider connection. (Scale bar = 1 mm)

**Video 2** Side-to-side anastomosis by using a silicone tube in the intraluminal continuous suturing technique. (Scale bar = 1 mm)

**Video 3** End-to-side anastomosis by using a silicone tube in the intraluminal continuous suturing technique. (Scale bar = 1 mm)

**Video 4** End-to-end anastomosis by using a silicone tube in the intraluminal continuous suturing technique. (Scale bar = 1 mm)
